# Supplementary material for: Protonic solid-state electrochemical synapse for physical neural networks
Source: Nat Commun. 2020 Jun 19;11:3134. doi: 10.1038/s41467-020-16866-6 (PMC7371700; doi:10.1038/s41467-020-16866-6)
Supplement: Supplementary file 1 — Supplementary Information [file 41467_2020_16866_MOESM1_ESM.pdf]

Supplementary information:

Protonic Solid-State Electrochemical Synapse for Physical Neural Networks

Xiahui Yao[1], Konstantin Klyukin[2], Wenjie Lu[3], Murat Onen[3], Seungchan Ryu[4], Dongha Kim[2], Nicolas Emond[2], Iradwikanari Waluyo[5], Adrian Hunt[5], Jesús A. del Alamo[3,*], Ju Li[1,2,*] and Bilge Yildiz[1,2,*]

1. Department of Nuclear Science and Engineering, Massachusetts Institute of Technology, 77 Massachusetts Avenue, Cambridge, Massachusetts, 02139, USA

2. Department of Materials Science and Engineering, Massachusetts Institute of Technology, 77 Massachusetts Avenue, Cambridge, Massachusetts, 02139, USA

3. Department of Electrical Engineering and Computer Science, Massachusetts Institute of Technology, 77 Massachusetts Avenue, Cambridge, Massachusetts, 02139, USA

4. Department of Mechanical Engineering, Massachusetts Institute of Technology, 77 Massachusetts Avenue, Cambridge, Massachusetts, 02139, USA

5. National Synchrotron Light Source II, Brookhaven National Laboratory, Upton, New York, 11973, USA

[*] byildiz@mit.edu, liju@mit.edu, alamo@mit.edu

Supplementary figures:

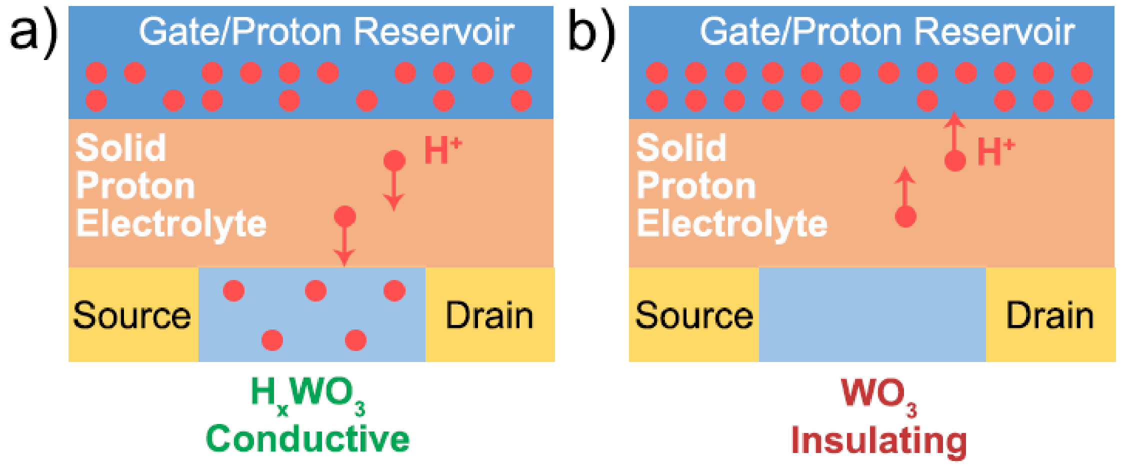

Supplementary Figure 1. a) Positive gating current applied on the gate allows proton intercalating into the channel and increases its conductance. b) Reversed polarity of the gate current de-intercalates the protons from the channel and reduces its conductance.

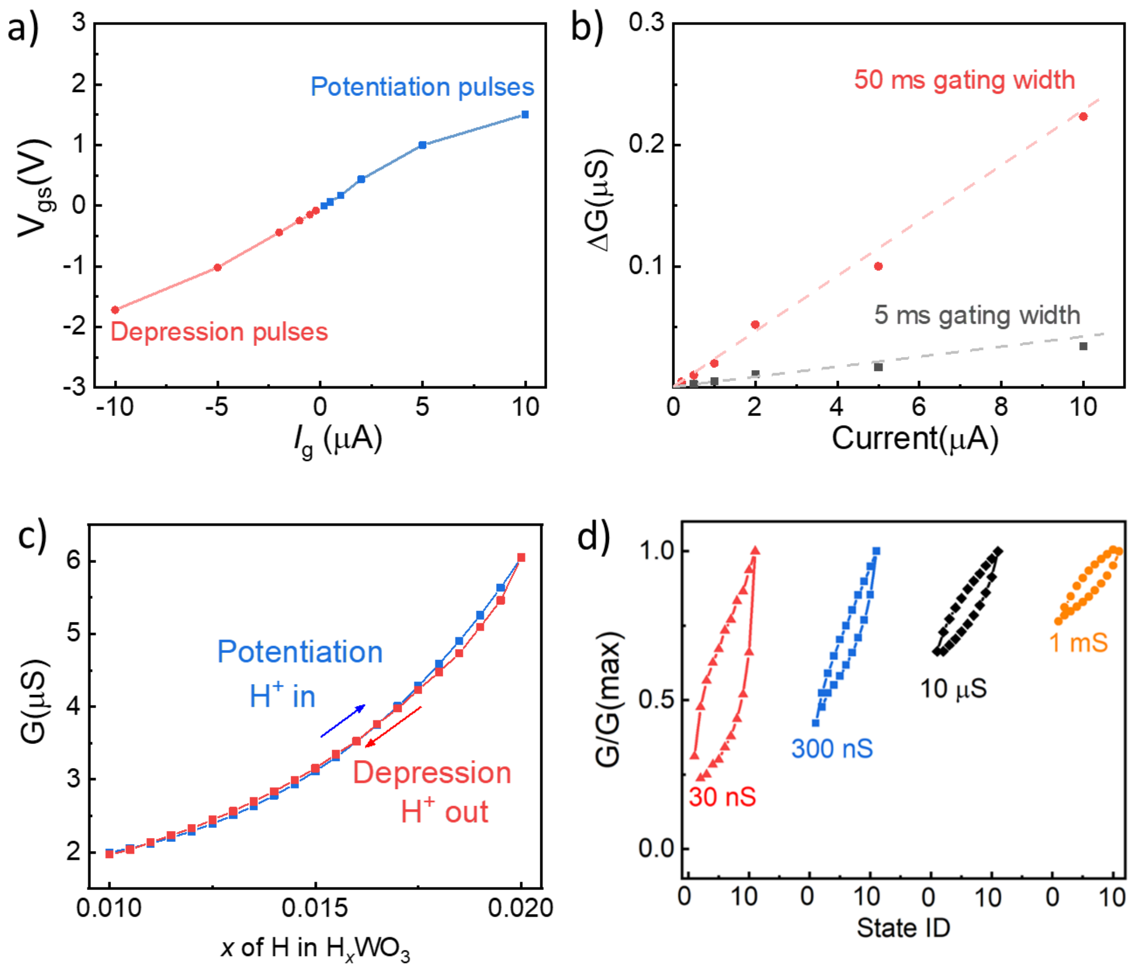

Supplementary Figure 2. a) Dependence of gating voltage on gating current with 5 ms pulsing width. The most energy consuming operation in a hardware neural network is the programing of the weights.[1] This is especially true when the programming process involves the movement of large cations or anions as in the conductive filament mechanism, or Joule heating in the phase change mechanism.[2] Our device is characterized by a small gating voltage.

b) Dependence of channel conductance change on different gating current with 5 ms or 50 ms constant current pulse width. In this electrochemical synapse, the conductance change of the active material depends on the amount of injected charge (Q).[3] This is determined by the integration of gate current over time. Constant-voltage bias typically generates a non-linear current response, which depends on the kinetics of the ion motion and the relative difference between the biasing voltage and the equilibrium chemical potential. On the other hand, the constant-current mode provides a linear relationship between Q and t, eliminating the need to keep track of the Open

Circuit Potential (OCP) as in the constant gate voltage pulse mode. This linearity enables the rational design of gating protocols to achieve the desired potentiation/depression behavior of the electrochemical synapse.

c) Good symmetrical operation when operated in the lower conductance regime of the demonstrated device. Each data point represents a stable conductive state after a protonating or de-protonating pulse (20 potentiation + 20 depression).

d) Comparison of 10 states at different conductance level. The four color represent different starting conductance level. Red: 30 nS, Blue: 300 nS, Black: 10 μS, Orange: 1 mS. The conductance data of the ten states obtained with the 10 potentiation and 10 depression pulses are normalized to maximum conductance obtained in the process. The plot clearly shows that the same amount of proton result in difference in the G_{max}/G_{min} ratio at different conductance level. The area of the loop represents the hysteresis between the potentiation and depression.

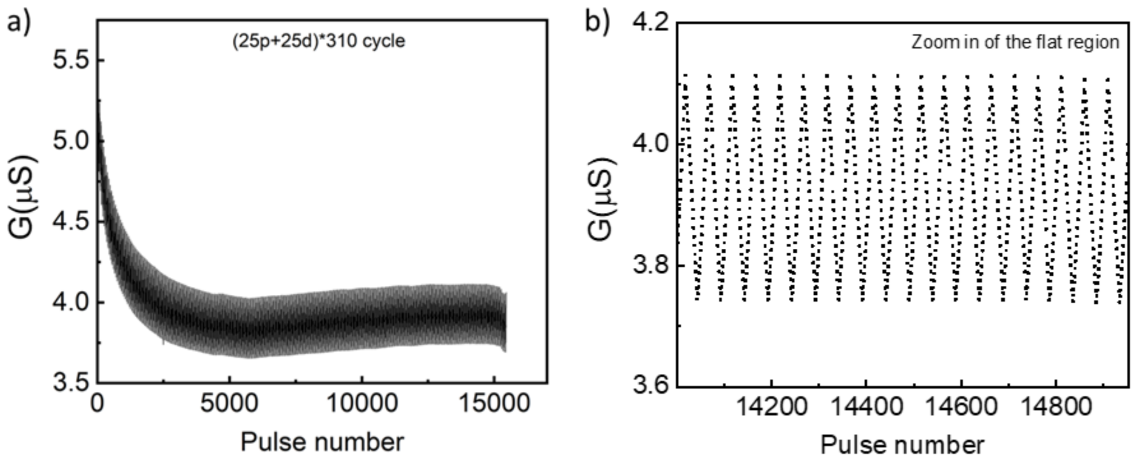

Supplementary Figure 3. a) Cycling behavior in the low conductivity region, with 25 potentiation pulses and 25 depression pulses for each cycle, lasting 310 cycles. The device is un-encapsulated. b) Zoomed in view of the flat region. Each dot represents the average conductance measured over the 1 second period between two pulses.

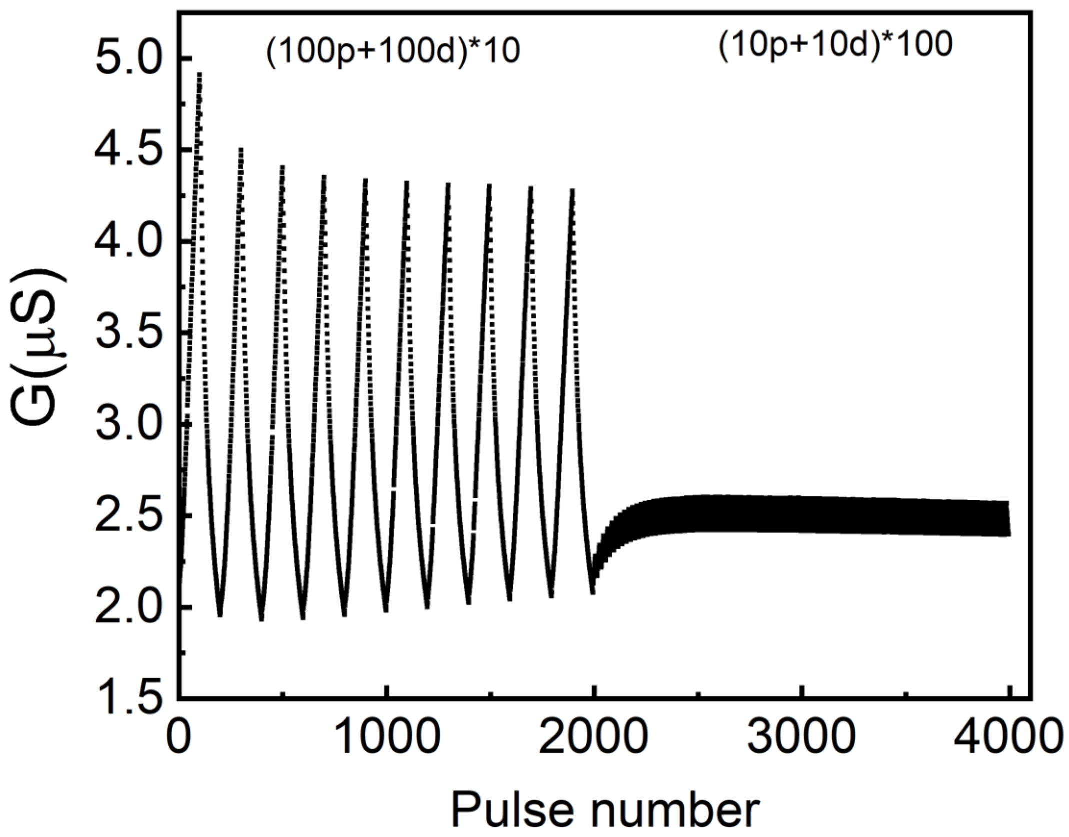

Supplementary Figure 4. Cycling behavior at low conductivity region, with dependence of pulse number per cycle as noted on over the curve. This plot clearly shows the dependence of G_{max}/G_{min} on the pulse number used in the gating. Also, the endurance of the unencapsulated device is demonstrated again here.

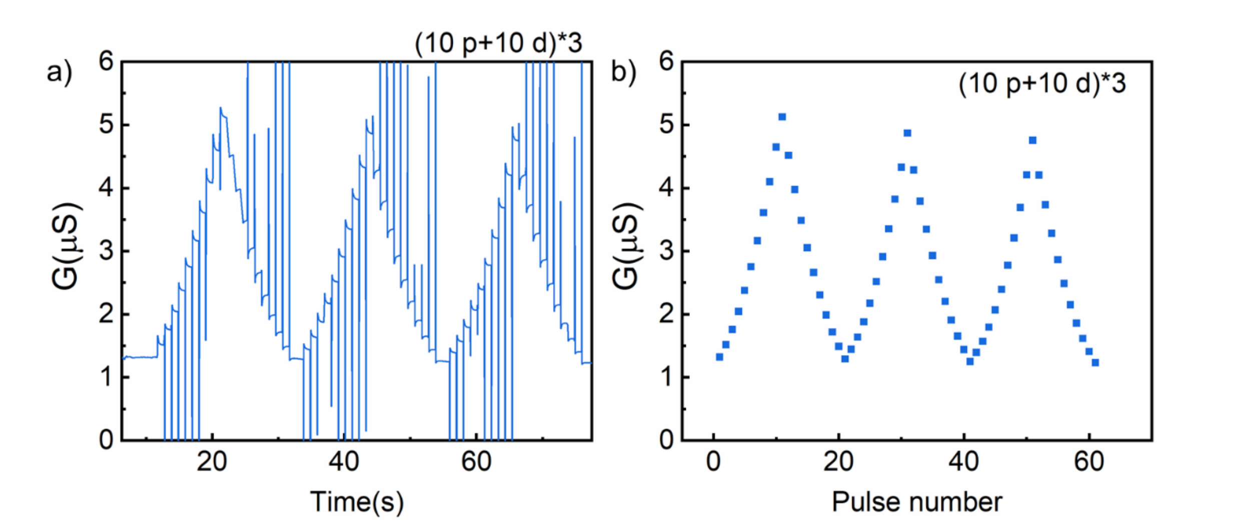

Supplementary Figure 5. Switching behavior of the device with WO_3 annealed at 400 °C for 1h instead of 50°C. a) The raw data of conductance *vs* real operating time, demonstrating the stable state after each pulse. b) Extracted states *vs* the pulse number to demonstrate the reversibility and low hysteresis of the cycling.

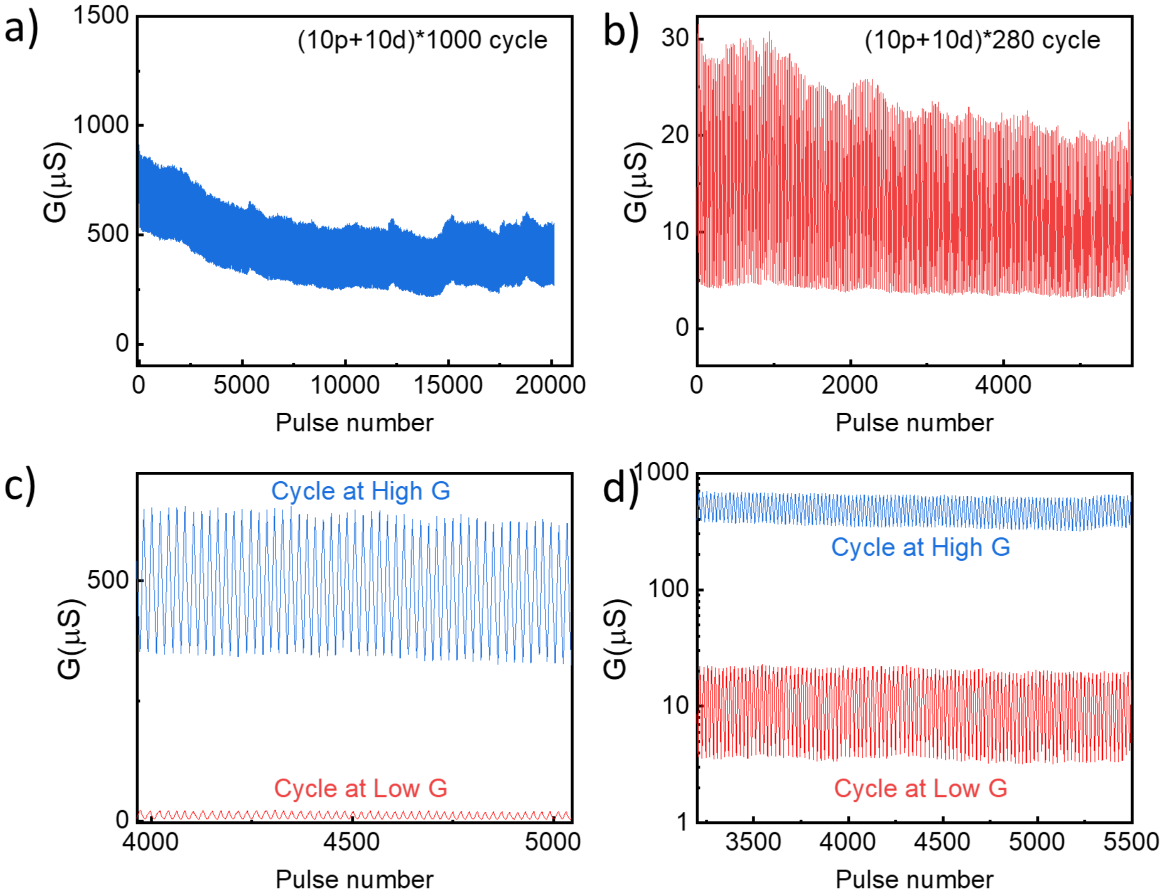

Supplementary Figure 6. Cycling behavior of encapsulated device, with dependence of pulse number per cycle as noted on over the curve. The device is encapsulated by UV-curable polymer and further covered by insulating epoxy after hydrogenation, to isolate the device from ingression of oxygen from ambient air. a) cycling at high conductance regime, b) cycling at low conductance regime, c) zoomed in view comparing the cycling in linear scale conductance. The amplitude of the pulsing current level (0.5 μA) and width (50 ms) are identical for these two regimes. d) Logarithmic scale of conductance of the zoomed in view of cycling data, with clear indication of G_{max}/G_{min} ratio being difference for the same amount of hydrogen insertion/remove in different conductance regimes.

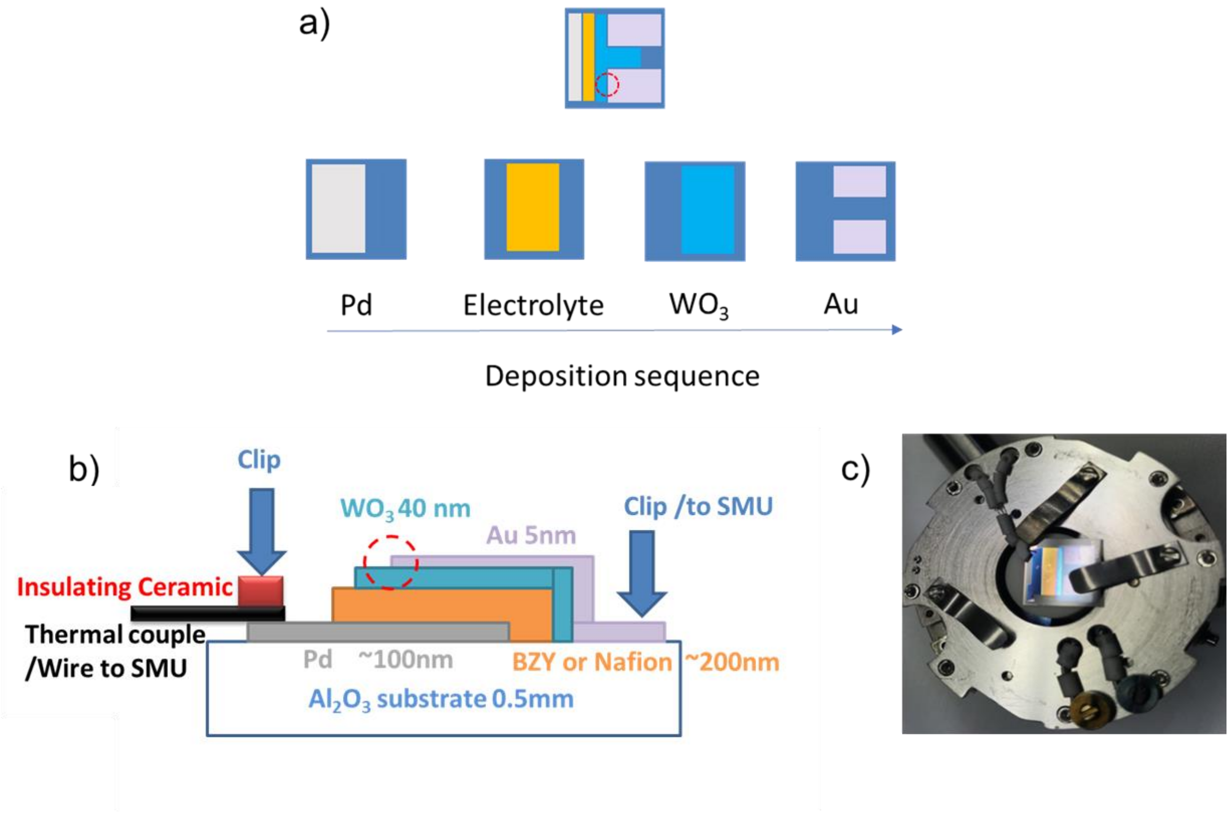

Supplementary Figure 7. a) The deposition sequence of the inversely ordered device for X-ray absorption and X-ray photoelectron spectroscopy measurements. b) Cross-sectional schematic of the device. The region for probing was noted as the red dashed circle. c) Picture of the test device mounted on the in-situ XAS sample holder with biasing capability before measurement.

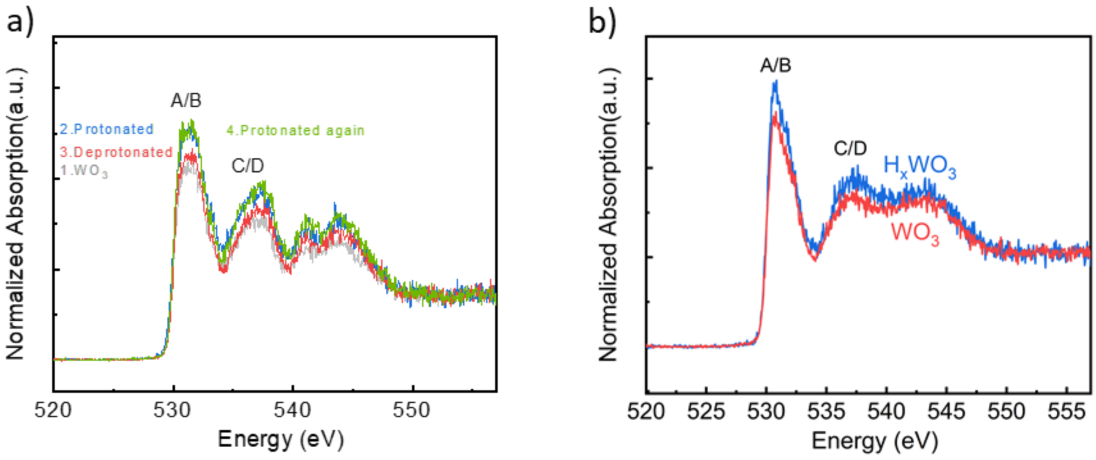

Supplementary Figure 8. O K-edge XAS measured on the WO_3 region with the following operation sequence:

1. WO_3: Measured before effective biasing, with H_2 and H_2O presence in the chamber
2. Protonated: Negative current was applied on the WO_3 (positive I_g on Pd gate electrode, +10 µA, ~15min), measurement is made after bias is removed.
3. Deprotonated: Positive current was applied on the H_xWO_3 (negative I_g on Pd gate electrode -40 µA, ~15min), measurement is made after bias is removed
4. Protonated again: Negative current was applied on the WO_3 (H_xWO_3) again, measurement is made after bias is removed

a) The solid electrolyte used in this sample was $BaZr_{0.8}Y_{0.2}O_3$, a well-known ceramic proton conductor. The biasing was operated at 340°C to compensate the low proton conductivity as a result of the low water and hydrogen vapor pressure allowed for *in operando* XAS system, in which p(H_2) = 400 mTorr, p(H_2O)=5 mTorr.

b) The sample with Nafion as electrolyte also exhibits the similar peak intensity change after protonation. Due to the inversed deposition sequence and thermal stability of Nafion, the WO_3 grown on sample b) was used in the as deposited form without the annealing step at 450 °C (amorphous WO_3).

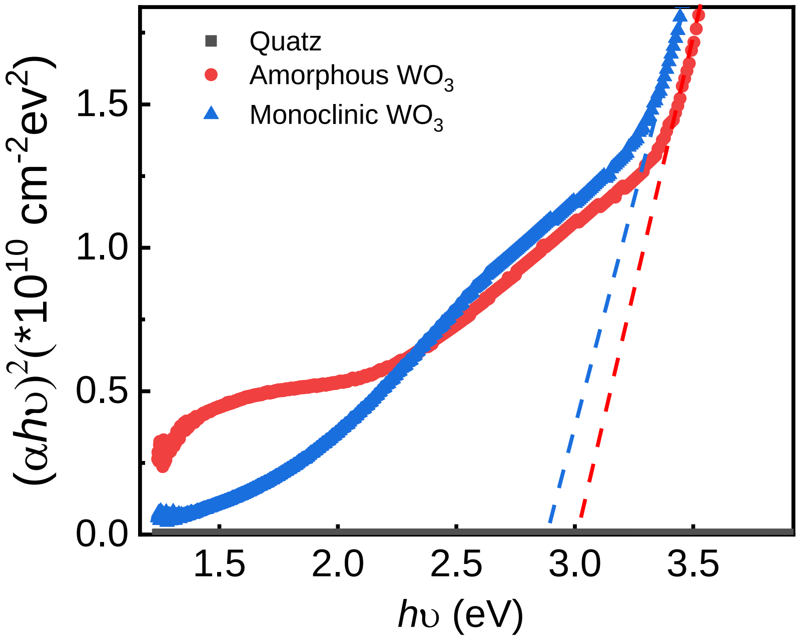

Supplementary Figure 9. Light absorption bandgap of WO_3 was measured to be from 2.8 eV to 3.0 eV with different crystal structure. The amorphous WO_3 was measured directly after deposition form reactive sputtering. The monoclinic WO_3 was obtained by further anneal the as-deposited sample in ambient air for 1 hour at 450 °C.

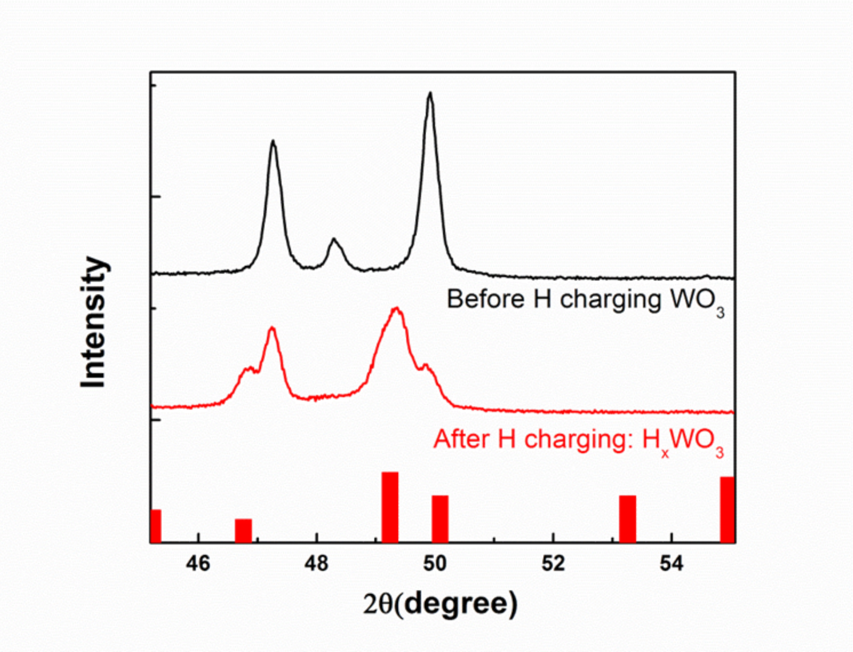

Supplementary Figure 10. High angle region of the XRD pattern comparing WO_3 and protonated H_xWO_3.

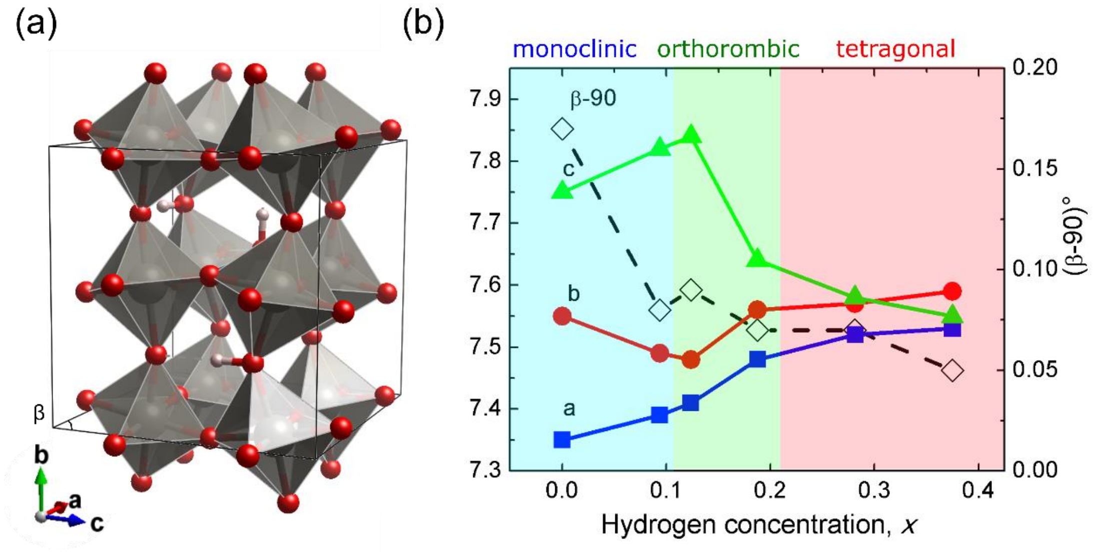

Supplementary Figure 11. Static DFT calculations (T=0 K) are employed to monitor evolution of WO_3 lattice parameters and β-angle upon hydrogen insertion. (a) Monoclinic unit cell of WO_3 was used to model the most energetically favorable configuration before hydrogen uptake (*x*=0). Tetragonal crystal structure of H_xWO3 was found stable at $x = 0.375$ assuming a homogeneous distribution of H over WO_3 lattice. Intermediate hydrogen concentrations were simulated by varying a partial occupancy of H pseudo atoms as implemented in VASP. (b) Evolution of structural parameters indicates that the interaxial angles approach 90° upon H insertion and transition between monoclinic and tetragonal phases occurs through orthorhombic phase. These results are in good qualitative agreement with our X-ray diffraction data shown in Figure 4 d and previous theoretical simulations on phase transformations caused by charge doping of WO_3.[4]

Computational Details. We used the hybrid functional of Heyd, Scuseria, and Ernzerhof corrected for solids (HSEsol), 4 × 4 × 4 Γ-centered k-point mesh and energy cutoff of 500 eV. All calculations are converged until the residual forces are below 0.04 eV/Å.

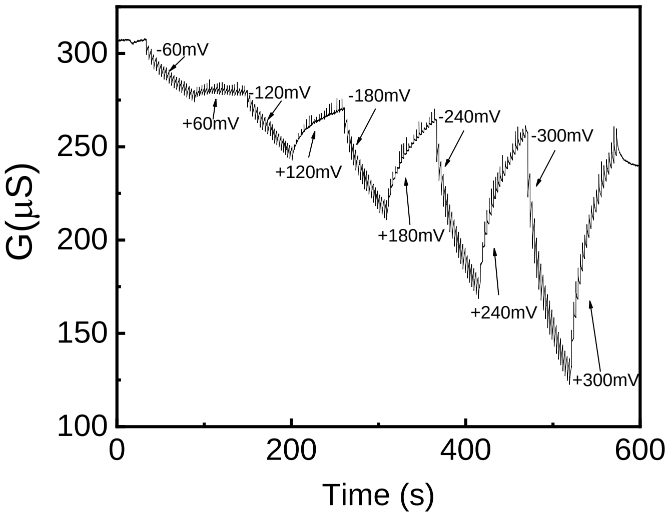

Supplementary Figure 12. Constant voltage gating of the channel WO_3 and corresponding conductance change. Positive or negative of the voltage level indicated on the plot was used to achieve the potentiation/depression behavior. With proper tuning of the voltage selected in the constant voltage gating, symmetrical switching can also be obtained. But it requires exploration of parameters specific to each device, or the same device at different state of charge (H content). In contrast, constant current pulsing neglects such variation, and only induces the fixed amount of protons with the same pulsing width. Therefore, although similar switching behavior can be achieved by constant voltage gating, constant current gating is still considered advantageous.

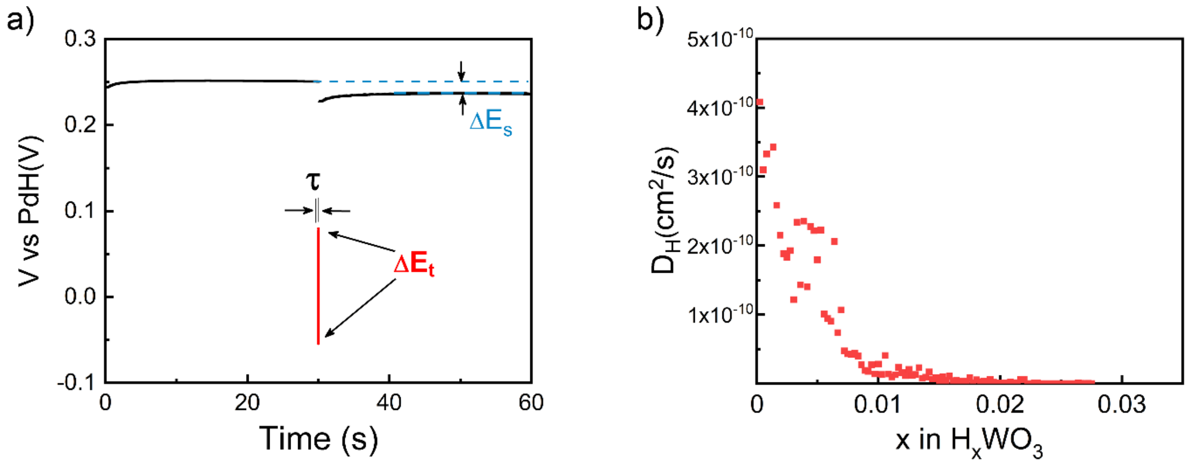

Supplementary Figure 13. a) Example data for one pulse (red trace) and the open circuit potential evolution (black trace) before and after the pulse in the Galvanostatic Intermittent Titration Technique (GITT) experiment. τ stands for the duration of each constant current pulse, ΔE_s is the steady-state chemical potential change as the result of the current pulsing, and ΔE_t reflects the voltage change during the constant current pulse. The voltage notation here is the channel (WO_3) vs the gate (PdH_x), reverse the value of the V_{gs}. b) The diffusivity of proton inside H_xWO_3 obtained from the GITT experiment in the low hydrogen content regime where x is between 0 to 0.03.

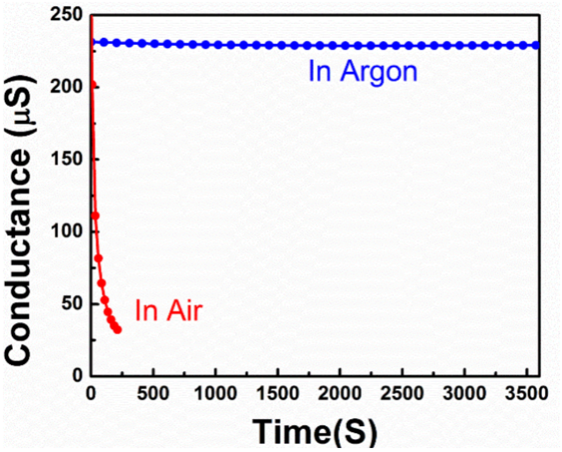

Supplementary Figure 14. Stability comparison of high conductance state of H_xWO_3 in air and in argon atmosphere. A quick degradation is observed when the unencapsulated device is exposed to air. A stable retention of the conductance is observed when the device is protected under argon inert atmosphere.

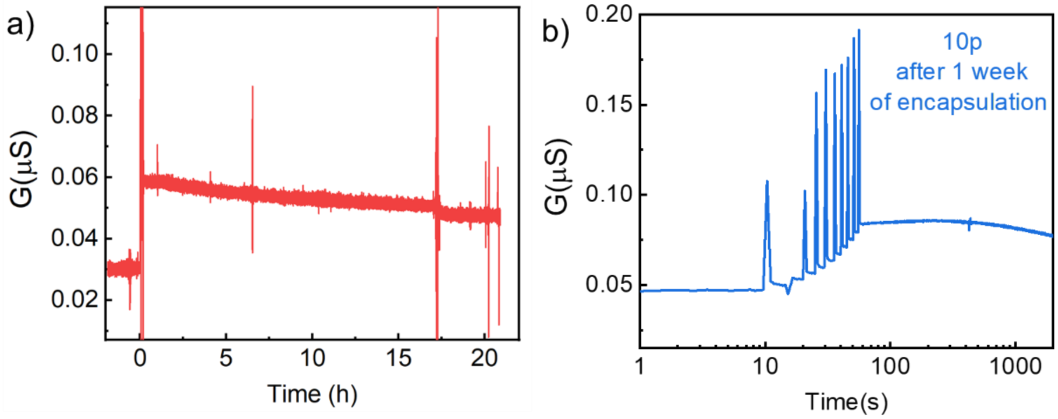

Supplementary Figure 15. Retention of a) an encapsulated device fresh after preparation with linear time scale and b) the encapsulated device after 1 week of preparation with log time scale. The increase of conductance was induced by a potentiating pulse train containing 10 constant current pulses with 200 nA amplitude and 50 ms width. The pulse train started at the time mark of 10 s. Between two pulses, the gating circuit was set as open circuit for 5 s, so that the channel conductance can be measured. After the pulse train, the gating circuits was opened again for long term retention measurement. The conductance of the channel is stable for tens of hours (a) and hundreds of seconds before degradation (b) demonstrating the robustness of the encapsulated device. Currently for our proof-of-concept device, the encapsulation approach is not ideal, and rather rudimentary. So any data that we collect with this approach will not represent the true potential of this device for endurance and retention. Characterization of the endurance and retention in properly encapsulated devices warrants future work.

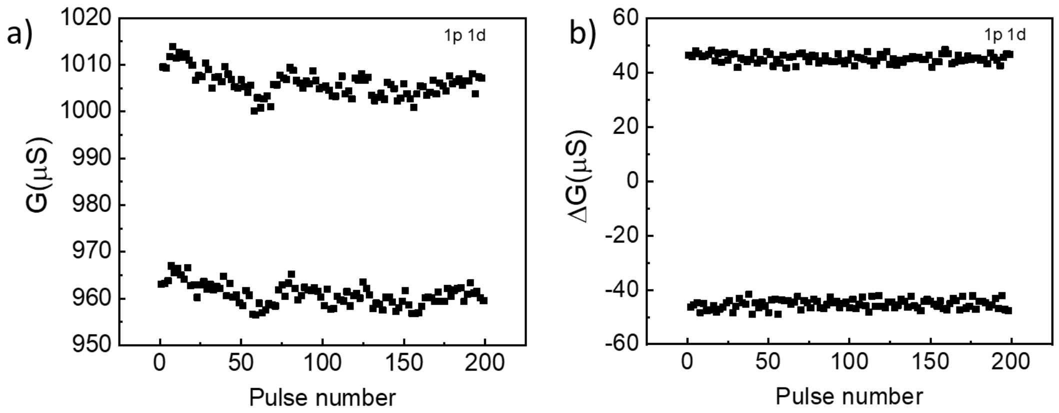

Supplementary Figure 16. a) Conductance (G) and b) conductance change (ΔG) with alternating 1 potentiation and 1 depression operation for 100 cycles. Reproducible switching behavior with low hysteresis is evident.

The switching behavior is more reversible with less proton exchanged for each cycle. We have demonstrated the good symmetry with reduced current density for each pulse in Figure 1d and the dependence of pulse number in each cycle in Figure 2b-d. This figure here provides additional information on pulse number dependence, where only 1 potentiation and 1 depression operation are used to induce the resistive behavior. Highly reproducible state change is evident here for 100 cycles. Each pulse has ±500 nA amplitude and 5 ms width.

Supplementary Table 1. Calculation of hydrogen content in WO_3 film during in-situ XRD

Sample label	Current (mA)	Time (s)	H (mol)	WO_3 (mol)	x in H_xWO_3
10 min	0.001	600	6.22×10^{-9}	1.28×10^{-7}	0.049
23 min	0.001	1380	1.43×10^{-8}	1.28×10^{-7}	0.112
54 min	0.001	3240	3.36×10^{-8}	1.28×10^{-7}	0.263

The dimension of WO_3 film: 0.826 cm × 0.5 cm × 100 nm.

Device configuration from bottom to top: Au/WO_3/Nafion/Pd.

Gate current bias: 1 uA.

Supplementary discussion 1: Gating strategy of electrochemical synapses

Gating by voltage signal is widely adopted in the literature to induce resistive switching due to its simplicity of implementation.[5] Typical reported gating voltage values range from several volts to tens of volts. Very often, the gating voltage used as a descriptor to indicate the final state of the sample.[6,7] Here we want to emphasize that, in such electrochemical three-terminal synapse devices, there can be a difference between the applied voltage and the equilibrium chemical potential. Since the gating process is essentially the charging of a capacitor, a good analogy is that the voltage to charge the capacitor (V_g here) and the voltage that the capacitor will retain (Open Circuit Potential, or chemical potential here) will be different before the capacitor is fully charged (sufficiently long charging time is allowed). And such difference could be one major reason for the lack of consistency of the gating voltages in the literature.[6,8-10] There are two major aspects to consider.

First, the voltage applied during the ionic gating is a dynamic voltage, not a steady-state chemical potential. Different from traditional electronic devices, in which only electrons or holes are mobile, the gating signal in the ionic device has to be converted into the flow of ions at the electrode-electrolyte interface. Therefore, the applied voltage contains three components: the chemical potential difference between the two electrodes, ionic ohmic loss across the electrolyte, and the Nernst overpotential at the electrode to drive the electron-ion exchange at the interface. For ionic gating, only the material status change, reflected by the chemical potential, is important to determine the channel conductivity. But to achieve the same amount of chemical potential change, the total applied voltage needed will be different across different material systems. This results in the scatter of gating voltage values that is observed in the literature. In addition, the absence of a clear indication of the reference for the applied voltage (the potential of electrochemical reaction at the counter electrode) is another major source of this inconsistency. Moreover, even for the same material system, the contribution from these three terms could be different at different conduction states of the material. All these factors result in the unpredictability of the gating voltage needed for switching, even for the same type of device. As an example, we adopted the constant voltage gating mode to induce the conductance change in Supplementary Figure 12. Although proper switching behavior can be induced, one cannot rationally design what the gating voltage should be, to achieve symmetrical switching behavior without trial and error for each different device.

Second, even if the biasing voltage-chemical potential correlation can be properly established, the time needed to reach the equilibrium between chemical potential on the surface and in the bulk is non-negligible. The mobile ions move from the electrolyte layer into the surface of the active material under the driving force of an electric field, and diffuse further into the bulk material to give rise to the change in the property of interest. The diffusivity of ions is typically much lower than electrons, in the order of 10^{-10}~10^{-15} cm^2/s. Therefore, the surface to bulk diffusion in the active material process is non-negligible and the time needed to reach equilibrium depends heavily on the diffusion coefficient and the sample thickness. This sluggish diffusion results in a difference between the applied biasing voltage and the final equilibrated chemical potential of the material if only short pulses are applied. Evidence can be found in Supplementary Figure 13a, in which -0.05 V voltage (vs PdH_x, this means V_{gs}= +0.05 V) is used to potentiate the channel for 5 ms, but the open circuit potential of the channel is only changing from 0.25 V to 0.23 V, this equilibrium potential of 0.23 V is significantly different from the applied bias of -0.05 V. This indicates that the biasing voltage is not a good parameter to describe what final status the device is modulated to. To achieve the device status defined by pulsing voltage, much longer biasing time than what has been reported in the literature (ms level) or smaller voltage difference compared with open circuit voltage will be necessary. This can be estimated from the diffusion time equation below, in which *t* stands for the time needed for diffusion to the length of *x* with the diffusion coefficient of *D*.

$$t \approx \frac{x^2}{2D}$$

Based on these analyses, we propose two directions to minimize the discrepancy. First, we should use constant current gating to obtain deterministic switching behavior. A direct descriptor for the channel status (conductivity) is the proton content in the channel. Regardless of the resulting gate voltage, the constant current gating always delivers a well-defined amount of charge into the active material. Since the correlation between proton concentration and material conductivity has been established (e.g. Figure 2a for WO_3), we can quantitatively change the status of the material conductance over a wide range simply by controlling the integral of the current with biasing time. This method is invariant to the detailed condition of the electrolyte, or the gate electrode chemical potential, as long as the gating voltage is within the electrochemical stability window of the electrolyte so the Coulombic efficiency is 100 % and there is not electrical break down.

Second, we should introduce cations with high diffusivity, such as protons, and reduce the dimension of the device. In our demonstration, the proton is intentionally selected as the charge carrier to provide fast diffusion kinetics. To provide evidence of this, we employed Galvanostatic Intermittent Titration Technique (GITT) to probe the diffusivity of proton in our device. This technique utilizes the time dependent relaxation of the open circuit voltage of the device upon the application of electrical gate pulses to quantify the diffusion coefficient of protons in WO_3. We analyze the data by the following simplified equation below[11]:

$$D = \frac{4}{\pi\tau}(\frac{n_m V_m}{S})^2(\frac{\Delta E_s}{\Delta E_t})^2$$

Here, τ stands for the duration of each constant current pulse, n_m is the quantity of material in moles, V_m is the molar volume of WO_3 (cm^3/mol), S represents contact area between WO_3 and the electrolyte, ΔE_s is the steady-state chemical potential change as a result of the current pulsing, and ΔE_t reflects the voltage change during the constant current pulse (Supplementary Figure 13a)

As demonstrated in Supplementary Figure 13b, the diffusion coefficient of H in H_xWO_3 is of the order of 10^{-10} cm^2/s at the initial protonation stage. This value correlates well with previous literature reports of proton diffusivity in WO_3 measured through liquid protonation.[12] An interesting observation is the reduction of D_H as the degree of protonation increases. This behavior is typically explained by the mechanism of stuffed diffusion channels, originating from protons percolating the fast diffusion channel inside the WO_3 lattice or saturating the grain boundary channel.[13] This implies the states at higher proton content need longer time to reach equilibrium, which can be reflected by the different operating symmetry shown in Figure 2. This observation further highlights the importance of proper selection of operating regime to achieve desired switching properties.

Supplementary discussion 2: Degradation mechanism and mitigation

In the unprotonated state, the low conductance state of WO_3 is very stable.[14] Basically, it is a thermodynamically and kinetically stable material without any known degradation mechanism at room temperature. On the contrary, if left as an open system in the presence of oxygen, the higher conductance states, H_xWO_3, are potentially unstable. H_xWO_3 can lose its conductive state through the following chemical reaction:

$$H_xWO_3+O_2=H_2O + WO_3$$

In fact, we do observe quick conductance degradation, within tens of seconds, if our unencapsulated device is exposed to ambient air (Supplementary Figure 14). However, H_xWO_3 itself is thermodynamically stable. This means that if not exposed to oxygen, the degradation of the material should be eliminated. Indeed, the devices kept under inert atmosphere showed long-term retention of the conductance (Supplementary Figure 14).

To demonstrate the long-term stability in a more realistic condition, we employed a polymer encapsulation approach to package our device. The encapsulated device can be tested in ambient air without the protective atmosphere. The cycling performance has been shown in Supplementary Figure 6 before and the retention of the state is shown below in Supplementary Figure 15. The device is still operable after weeks of encapsulation before optimization of the packaging approach, demonstrating the robustness of the device. We envision, with better sealing, better retention can be obtained.

Another potential degradation mechanism is the loss of protons as hydrogen gas or through diffusion into the substrate during the electrochemical cycling between the PdH_x and WO_3. As an electrochemical process, the Faradaic efficiency is desired to be 100% but the realistic efficiency is often less than ideal. We do not have any evidence of this issue in our experiments. However, such a challenge could show a cumulative effect over very large number of cycles.

Our strategy to mitigate this possible mode of degradation is through the redundant storage of hydrogen in the solid reservoir layer. Two factors are important for this: 1) The solid hydrogen reservoir layer should have a high hydrogen storage capability, and 2) A small concentration of protons should lead to a signification change of the conductance of the channel material. Regarding the first point, palladium is one of the best hydrogen storage materials since it can hold up to 1

hydrogen atom per unit Pd. Properly tuning the relative thickness ratio between the Pd and the WO_3 active layer can provide the desired hydrogen utilization fraction over the targeted life cycle of the device.

However, the thickness of hydrogen storage material involved should still be small. That brings forth the second point, a small amount of hydrogen should give rise to large enough modulations of conductivity. In order to serve as accurate accelerators in AI, the conductance range of the device should be at least one order of magnitude[15]. This device needs only as low as 1 mole percent of H to achieve one order of magnitude on/off ratio, as shown in low H content region in Figure 2a. Therefore, even if the Faradaic efficiency is less than 100%, we expect that the endurance of the device can still be satisfied.

Supplementary discussion 3: Dependence of Open Circuit Potential on the hydrogen content

Simultaneous transitions in the slope of the OCP between the channel (H_xWO_3) and gate (PdH_x) and the channel conductivity are seen in Figure 2a. This gives further evidence that the cause of transitions in the conductivity is likely related to a phase change in the channel material. As described in the Methods, we are operating this device (without an encapsulation) in a hydrogenated atmosphere for the evaluation of the intrinsic property of the active material. In this condition, the PdH_x gate electrode serves as a reliable reference electrode at open-circuit condition because it produces a stable chemical potential in contact with the Nafion when there is no net current. Therefore, the OCP measured here reflects the chemical potential of H_xWO_3 with respect to PdH_x. The change of the dependence of the chemical potential on x in H_xWO_3 indicates that different phases of H_xWO_3 are involved in the different regimes. Structural characterization presented in Figure 4d further support such phase transition.

Supplementary discussion 4: O:W stoichiometry

We used the W4f region to estimate the non-stoichiometry. The W 4 $f_{7/2}$ peak was deconvoluted into two peaks. The main peak was assigned to be W^{6+} and a very small shoulder peak was assigned to the reduced W due to oxygen vacancy. The relative percentage of these two peaks are 95.5% vs 4.5%. Based on the peak position, this reduced W has the oxidation state between W^{6+} and W^{5+}, if we assume them to be 5+ for the ease of calculation, we get the O:W ratio as 2.977. To be noted, this number is underestimated because the assumption we made, so we expect the true value to be higher than this and closer to 3.

Supplementary References

1. Deng, L. *et al.* Energy consumption analysis for various memristive networks under different learning strategies. *Phys. Lett. A* **380,** 903-909 (2016).
2. Yang, J. J. S., Strukov, D. B. & Stewart, D. R. Memristive devices for computing. *Nat. Nanotech.* **8,** 13-24 (2013).
3. Chua, L. Resistance switching memories are memristors. *Applied Physics A* **102,** 765-783 (2011).
4. Wang, W., Janotti, A. & Walle, C. G. V. d. Phase transformations upon doping in WO_3. *J. Chem. Phys.* **146,** 214504 (2017).
5. Sawa, A. Resistive switching in transition metal oxides. *Materials Today* **11,** 28-36 (2008).
6. Katase, T., Onozato, T., Hirono, M., Mizuno, T. & Ohta, H. A transparent electrochromic metal-insulator switching device with three-terminal transistor geometry. *Sci. Rep.* **6,** 25819 (2016).
7. Shibuya, K. & Sawa, A. Modulation of Metal–Insulator Transition in VO_2 by Electrolyte Gating-Induced Protonation. *Adv. Electron. Mater.* **2,** 1500131 (2016).
8. Grey, P. *et al.* Solid State Electrochemical WO_3 Transistors with High Current Modulation. *Adv. Electron. Mater.* **2,** 1500414 (2016).
9. Leng, X. *et al.* Insulator to metal transition in WO_3 induced by electrolyte gating. *npj Quantum Mater.* **2,** 35 (2017).
10. Yang, J.-T. *et al.* Artificial Synapses Emulated by an Electrolyte-Gated Tungsten-Oxide Transistor. *Adv. Mater.* **30,** 1801548 (2018).
11. Zhu, Y. & Wang, C. Galvanostatic Intermittent Titration Technique for Phase-Transformation Electrodes. *J. Phys. Chem. C* **114,** 2830-2841 (2010).
12. Randin, J. P. & Viennet, R. Proton Diffusion in Tungsten Trioxide Thin Films. *J. Electrochem. Soc.* **129,** 2349-2354 (1982).
13. Vértes, Á. & Schiller, R. Concentration-dependent diffusivity: Hydrogen percolation in WO_3. *J. Appl. Phys.* **54,** 199-203 (1983).
14. Ramana, C. V., Utsunomiya, S., Ewing, R. C., Julien, C. M. & Becker, U. Structural Stability and Phase Transitions in WO_3 Thin Films. *J. Phys. Chem. B* **110,** 10430-10435 (2006).
15. Gokmen, T. & Vlasov, Y. Acceleration of Deep Neural Network Training with Resistive Cross-Point Devices: Design Considerations. *Front. Neurosci.* **10,** 333 (2016).
